# Transcatheter arterial chemoembolization improves the resectability of malignant breast phyllodes tumor with angiosarcoma component: a case report

**DOI:** 10.1186/s12893-019-0562-0

**Published:** 2019-07-27

**Authors:** Chih-Yu Kuo, Shing-Huey Lin, Kuan-Der Lee, Sho-Jen Cheng, Jan-Show Chu, Shih-Hsin Tu

**Affiliations:** 1 Department of Surgery, Taipei Medical University Hospital, Taipei, Taiwan; 2 Division of Family Medicine, Cathay General Hospital, Taipei, Taiwan; 3 Division of Breast Surgery, Department of Surgery, Taipei Medical University Hospital, Taipei, Taiwan; 4 Department of Surgery, School of Medicine, College of Medicine, Taipei Medical University, Taipei, Taiwan; 5 Taipei Cancer Center, Taipei Medical University, Taipei, Taiwan; 6 Division of Hematology and Oncology, Department of Internal Medicine, Taipei Medical University Hospital, and School of Medicine, College of Medicine, Taipei Medical University, Taipei, Taiwan; 7 Department of Medical Imaging, Taipei Medical University Hospital, Taipei, Taiwan; 8 Department of Pathology, School of Medicine, College of Medicine, Taipei Medical University, Taipei, Taiwan

**Keywords:** Malignant phyllodes tumor, Angiosarcoma, Transcatheter arterial chemoembolization, Embozene microspheres

## Abstract

**Background:**

A giant phyllodes tumor of the breast is a rare fibroepithelial lesion, and its treatment is controversial. Many case reports have reported performing skin graft reconstruction after tumor excision. Chest wall resection may be required if the tumor has invaded the chest muscle layer. We speculated that transcatheter arterial chemoembolization (TACE) can improve the resectability of malignant phyllodes tumor of the breast without requiring skin grafting. The English literature contains only one case report similar to our experience.

**Case presentation:**

We report a rare case of a 51-year-old woman who had a giant malignant phyllodes tumor with heterologous sarcomatous differentiation in her right breast. The tumor was 19.43 × 12.98 × 21.47 cm. Whole-body computed tomography (CT) and bone scan did not reveal distant metastasis. Chest magnetic resonance imaging showed chest wall tumor invasion. Considering that skin defects after mastectomy can be extensive, we administered four courses of chemoembolization in the 5 weeks before surgery (30 mg of epirubicin and embozene microspheres [400, 500, and 700 μm]/week). Each process was well tolerated, with no serious complications. Only fever and local pain at the tumor site were noted, and these symptoms resolved with time. The follow-up CT scan showed a 45% reduction in tumor volume. Therefore, simple mastectomy was performed without skin grafting reconstruction. Wound healing was satisfactory, and the patient was discharged 1 week after surgery. Pathological and immunohistochemistry (IHC) findings showed a malignant phyllodes tumor with an angiosarcoma component. Because of tumor invasion of the chest wall, we recommended the patient receive radiotherapy, but she refused. Two months after surgery, recurrence of the malignant phyllodes tumor with right axillary lymph node involvement and lung metastasis was confirmed.

**Conclusion:**

Initial surgical resection of giant phyllodes tumors is often challenging. For initial presentation with unresectable giant phyllodes tumor, we recommend to perform TACE prior to surgery. In our patient, preoperative TACE was effective and safe. If the tumor has invaded the chest wall, early radiotherapy after surgery may be recommended for preventing recurrence.

## Background

Phyllodes tumor of the breast is a rare fibroepithelial lesion composed of both stromal and epithelial components, accounting for 0.3–1.0% of breast tumors [1]. Some of these tumors may grow rapidly and reach a large size (> 10 cm in diameter). After resection of the primary tumor, the reconstruction generally includes skin grafting, which involves harvesting of skin from another body part of the patient [2]. However, skin grafts may cause disease or adverse effects in patients, especially patients with large skin defects or contaminated wounds. Increased size and complexity of chest wall resections could require advanced reconstructive techniques.

TACE is performed by an interventional radiologist to restrict the blood supply of a tumor to cause tumor necrosis and shrinkage. Several studies have shown that preoperative tumor shrinkage has a good prognosis for advanced breast cancer metastasis [2–4]. Here, we report a case of a patient with a giant malignant phyllodes tumor that was successfully reduced through preoperative TACE. In the English literature, only one case has been reported that is similar to our experience. Hashimoto et al. (2016) reported on the preoperative chemoembolization of a large malignant phyllodes tumor with successful avoidance of skin grafting after excision [2].

## Case presentation

**Fig. 1:**
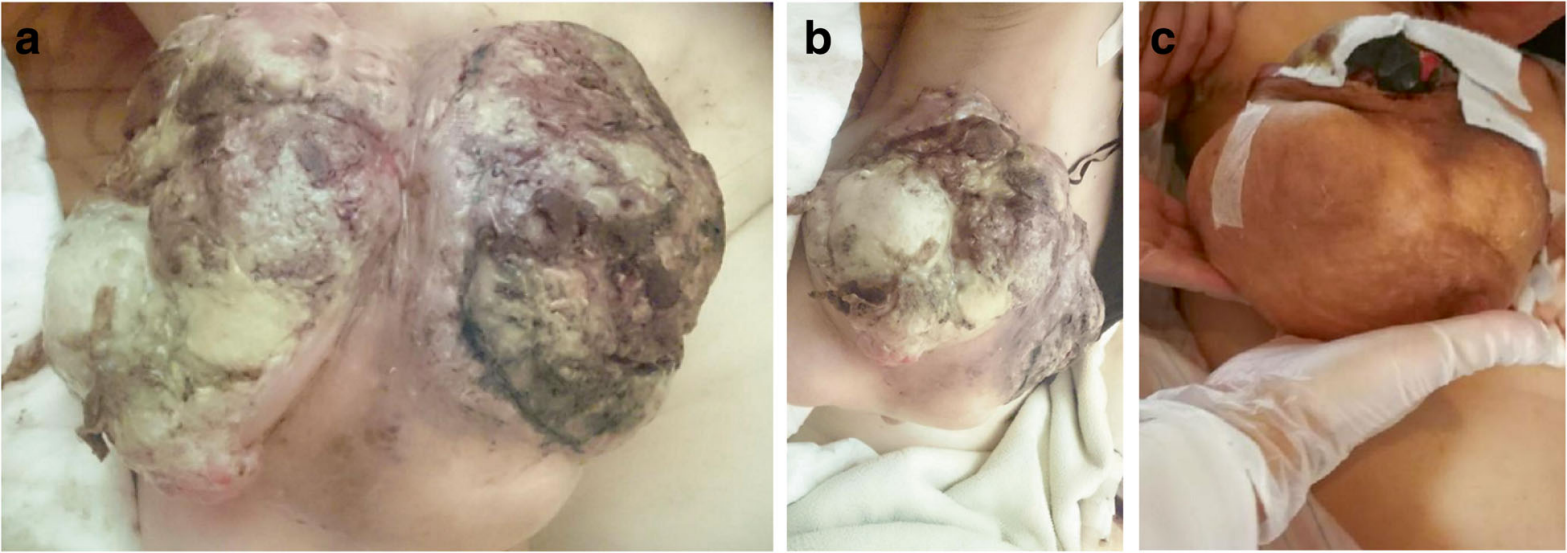
**a** Gross tumor before treatment. Entire breast surface revealed a fungating wound and necrotic changes. **b** and **c** Shrinkage of the gross tumor observed after four cycles of preoperative chemoembolization

A 51-year-old woman without any underlying systemic disease presented with a 1-year history of a bulky tumor in her right breast. She denied having any breast cancer history in her family. The tumor grew rapidly with a large fungating wound and necrotic edges (Fig. 1). She denied having undergone any treatment before the presentation. She was found to have an elevated white blood cell count (11.420/mm^3^) and a normal tumor marker levels of carcinoembryonic antigen (CEA) and carcinoma antigen 15–3 (CA153). She was hospitalized in our breast medical center and underwent surgical debridement. Antibiotics were administered to control the infection.

**Fig. 2:**
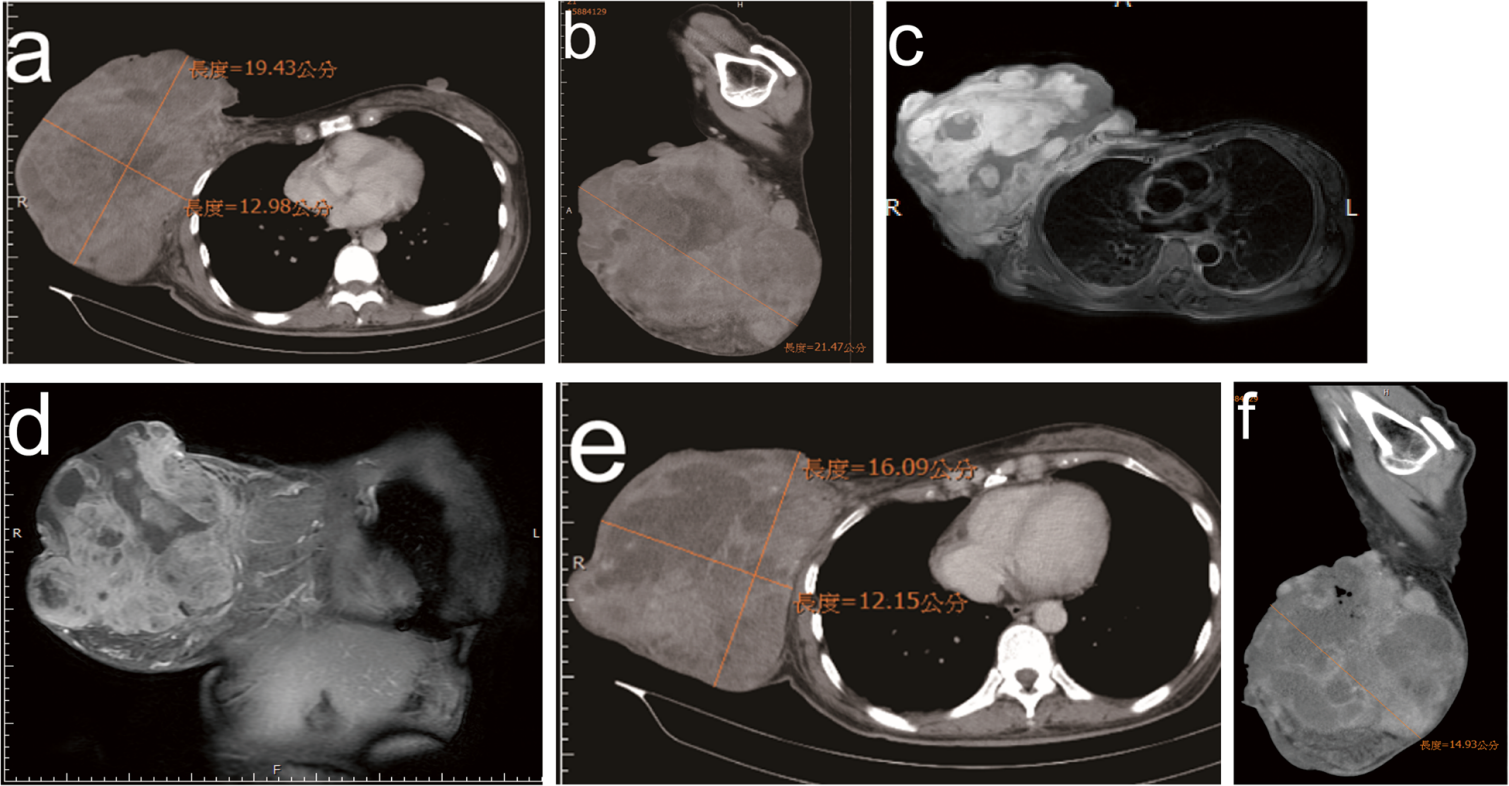
**a** and **b** Contrast-enhanced chest CT showed a huge lobulated heterogeneously enhancing mass (measuring approximately 19.43 × 12.98 × 21.47 cm) in the right breast. **c** and **d** Contrast-enhanced MRI showed a 20-cm lobulated right breast mass with right major pectoralis muscle invasion. **e** and **f.** After four courses of TACE, follow-up CT revealed tumor reduction of 45% of total volume (measuring approximately 16.0 × 12.0 × 15.0 cm)

Histological examination performed using a core needle biopsy (CNB) showed a spindle cell lesion that was positive for cluster of differentiation 34, hematopoietic progenitor cell antigen (CD34) and negative for beta-catenin, cytokeratin (CK), S100 calcium binding protein (S100), and signal transducer and activator of transcription 6 (STAT6). Because CK staining was negative, metaplastic carcinoma was unlikely. A definite diagnosis could not be established based on CNB specimen findings. Breast sonography revealed a huge heterogeneous mass. Breast CT showed a huge lobulated heterogeneously enhancing mass, measuring approximately 19.43 × 12.98 × 21.47 cm, in the right breast (Fig. 2a and b). No evidence of distant metastasis was observed with whole-body CT. A bone scan revealed no evidence of skeletal metastasis. Magnetic resonance imaging (MRI) revealed major pectoralis muscle invasion and several enlarged lymph nodes in the right axillary region (Fig. 2c and d).

Considering that the patient’s breast tumor is inoperable, because the entire chest wall tissue diffuse necrosis and skin defect after mastectomy may over 30 × 25 cm, we performed preoperative intra-arterial infusion chemotherapy and embolization to shrink the tumor before mastectomy. Four cycles of 30 mg of epirubicin plus embozene microspheres were administered with a 1-week interval between cycles (Table 1).

**Table 1:** Diameter of Embosphere Microspheres, Epirubicin used and the embolization vascular in each procedure

	The diameter of Embosphere Microspheres (micron)	Epirubicin (mg)	The embolization vascular
First procedure	400, 500	30	Right internal mammary artery
Second procedure	400	30	Thoracoacromial artery
			Lateral thoracic artery
			Thoracodorsal artery
Third procedure	400, 500, 700	30	Right internal mammary artery
			Lateral thoracic artery
			Thoracodorsal artery
Forth procedure	400, 700	0	Right internal mammary artery
			Thoracodorsal artery

^*^Embozene microsphere embolizers (400, 500, 700 μm) were selected for all treatments and diluted with a nonionic contrast medium and saline.

**Fig. 3:**
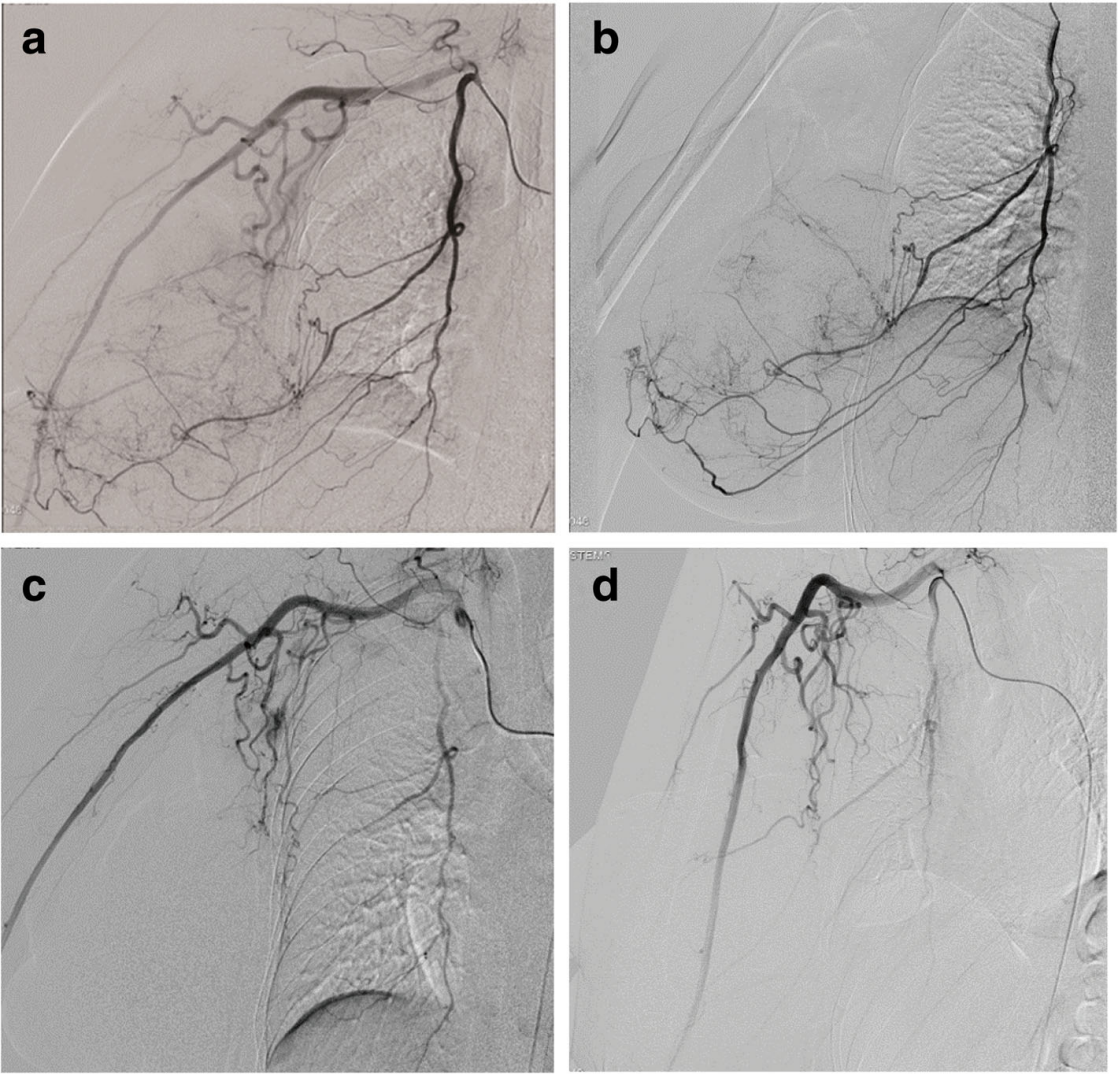
Arteriography identified the vascular blood supply of the tumor. **a** Right subclavian artery. **b** Internal mammary artery. **c** Lateral thoracic and thoracodorsal arteries. **d** Post-TACE showed all target tumor blood supply vessels

For chemoembolization, after the administration of local anesthesia, a angiosheath was inserted into the right femoral artery, followed by the introduction of an angiocatheter and a microcatheter. The vascular blood supplies of the tumor, right internal mammary artery, thoracoacromial artery, lateral thoracic artery, and thoracodorsal artery were identified (Fig. 3). A pulsed injection of 30 mg of epirubicin was administered into the tumor through the microcatheter. Subsequently, embozene microspheres were injected.

**Fig. 4:**
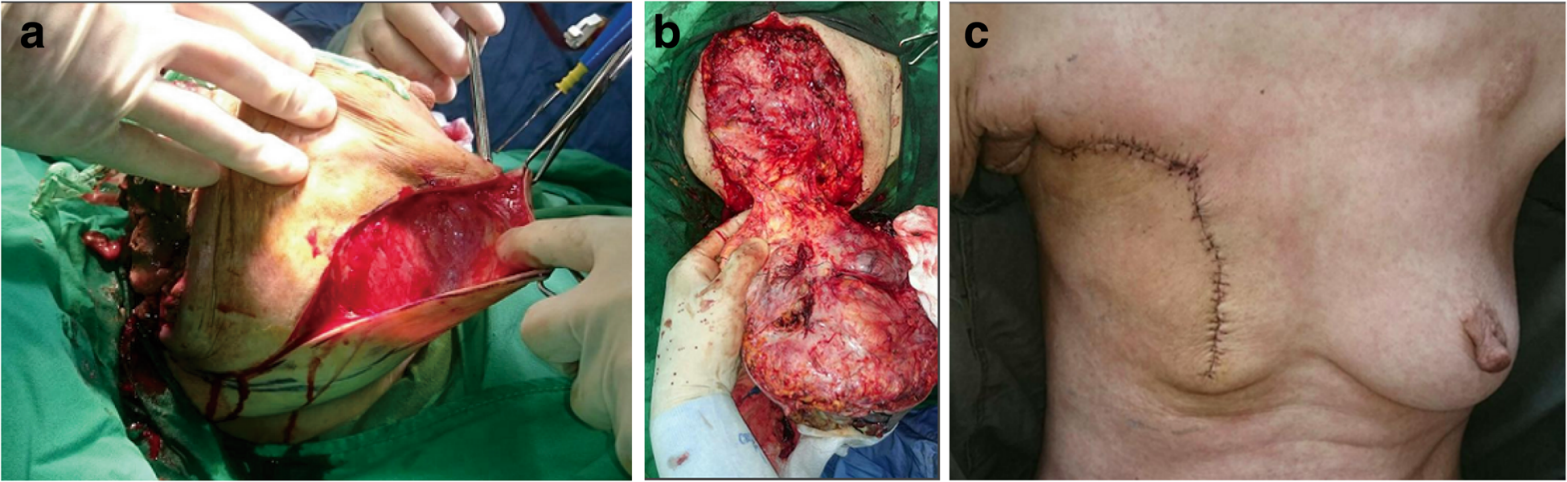
**a** and **b** Simple mastectomy was performed. **c** After surgery, the tumor was completely resected without skin grafting

After chemoembolization, fever and right chest pain at the tumor site were noted. Symptoms resolved within 2 days. No other serious complications were observed after TACE. Follow-up CT revealed a 45% reduction in total tumor volume (Fig. 2e and f). Next, simple mastectomy was performed. Skin grafting was not performed during surgery (Fig. 4). The tumor-free margin was reported after further pathological examination.

The right breast measured 26 × 20 × 10 cm in size and 1835 g in weight. Pathological findings indicated the presence of a malignant phyllodes tumor with an angiosarcoma component. The surgical margin was not involved by the tumor. In addition, the nipple and axillary nodes were not involved by the tumor.

The surgery successfully treated tumor pain and caused tumor necrosis. Because the tumor invaded the chest wall muscle layer, we speculated that concerning microscopic foci could have remained. Therefore, we recommended the patient undergo adjuvant irradiation to prevent recurrence. However, the patient rejected this therapeutic option and was lost to follow-up for 2 months.

When the patient presented again 2 months after surgery, we noted the development of a palpable mass over the right chest wall. A palpable lymph node was observed in the right axillary region. Follow-up CT showed a mass measuring 5.0 × 4.0 cm over the right chest wall and a palpable lymph node measuring 6.0 × 5.0 cm was found in the right axillary region, beneath the pectoralis muscle. Additionally, several ground glass lesions were noted in the bilateral lungs. CNB findings confirmed lymph node metastasis. Therefore, malignant phyllodes tumor recurrence with axillary lymph node involvement and lung metastasis was diagnosed. Furthermore, pathology and immunohistochemistry (IHC) findings confirmed the presence of a malignant phyllodes tumor with recurrence. The patient obtained four courses of additional chemotherapy and two fractions of radiotherapy after recurrence was found (2 months after surgery). After that, she refused further treatment and was lost to follow-up.

## Discussion and conclusions

CNB is widely used as a highly sensitive method for obtaining a preoperative diagnosis of breast cancer [5]. However, diagnostic challenges in pathological interpretation and controversies regarding the management of certain lesions diagnosed through percutaneous CNB remain. Although histological features, IHC findings, and genetic test results can help establish a diagnosis, the overlap of pathological features and small amount of the tissue obtained in CNB cause difficulty in the diagnostic classification of these lesions [6].

In our case, the pathological findings of the first CNB specimen revealed spindle tumor cell proliferation with mild atypia on the dense collagenous stromal background. Diagnosis of a spindle cell lesion in the breast is particularly difficult when encountered in CNB findings. Several diagnoses should be considered for a spindle cell lesion of the breast. Because of our patient’s clinical appearance, the presence of a malignant lesion was highly suspected, including a malignant phyllodes tumor, metaplastic carcinoma, melanoma, angiosarcoma, or other primary breast sarcoma [7].

**Fig. 5:**
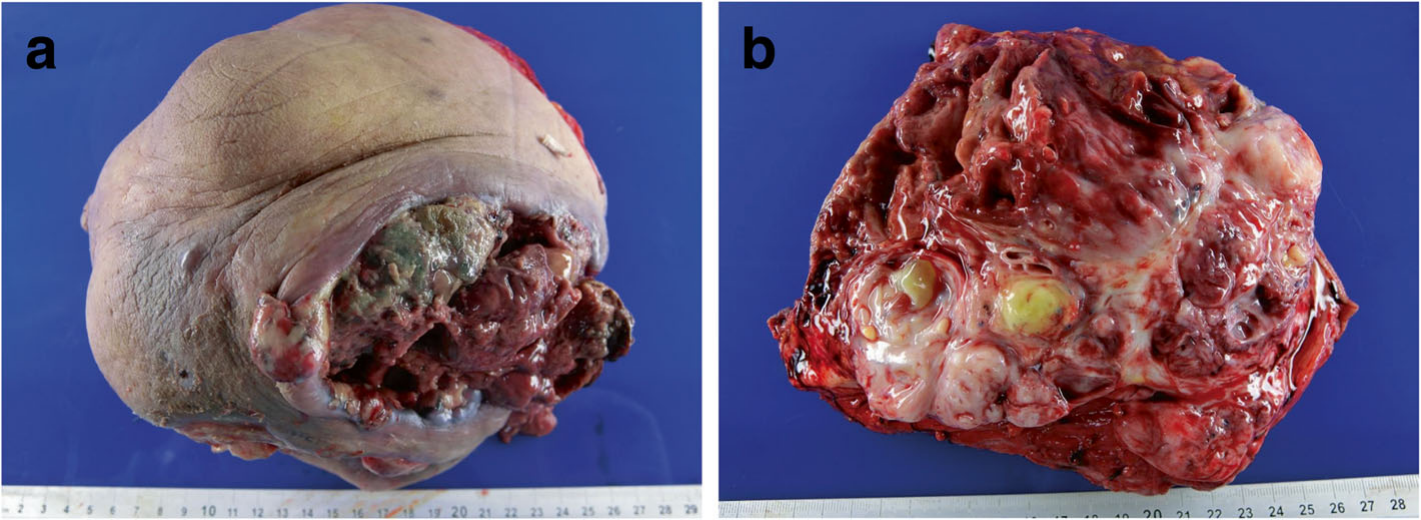
**a** The right breast measured 26.0 × 20.0 × 10.0 cm. The skin was covering it elliptical in shape with ulcerative changes. **b** Anastomosing and dissecting vascular channels were found

**Fig. 6:**
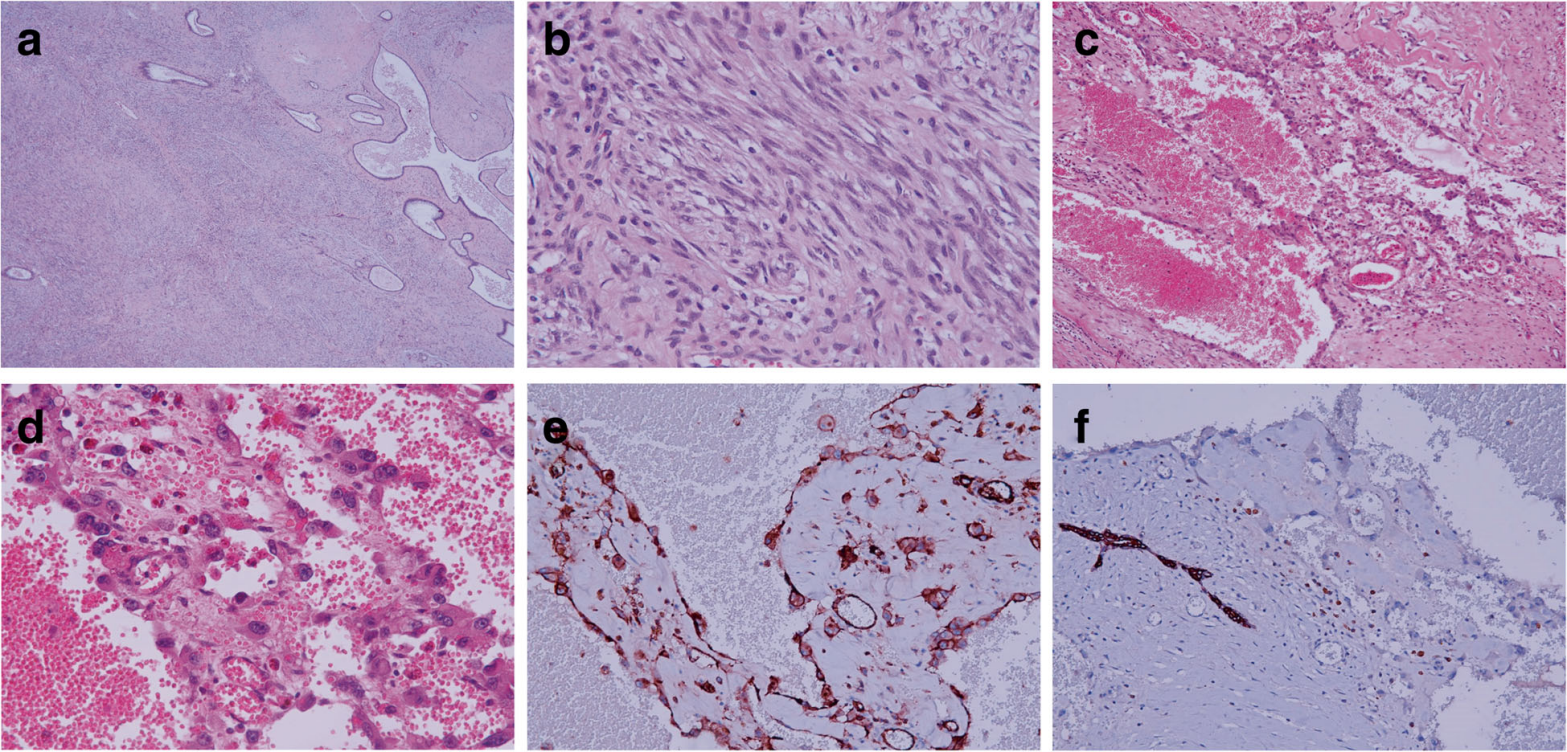
**a** Stromal component showed leaf-like appearance. **b** Stromal overgrowth with a spindle cell feature. **c** and **d** Atypical cells in the vascular space. **e** Vascular endothelium present with positive CD31. **f** CK negative in the vascular space, CK staining positive at the ductal epithelium

Distinguishing metaplastic carcinoma and malignant phyllodes tumors of the breast is critical because their treatment and prognosis differ significantly. The leaf-like architecture and absence of CK expression can be helpful markers of phyllodes tumor [8]. The pathological findings of our case after surgery showed a leaf-like structure (Fig. 6a). The stromal component revealed increased cellularity, moderate nuclear pleomorphism, increased mitotic activity, and stromal overgrowth (Fig. 6b). All these pathological features are often observed in malignant phyllodes tumors. IHC is a useful tool in the diagnosis of a spindle cell lesion. In our case, IHC results were positive for CD34 but negative for beta-catenin, CK (AE1/AE3), S-100, and STAT6. Although no marker is perfectly sensitive or specific, the majority of phyllodes tumors are positive for CD34, and CD34 expression is seldom seen in spindle cell metaplastic carcinoma. Anastomosing and dissecting vascular channels lined by flat atypical cells in the vascular space were observed (Fig. 5c and d). Pleomorphic cells are positive for cluster of differentiation 31, platelet endothelial cell adhesion molecule (CD31) (Fig. 6e) and erythroblast transformation-specific related gene (ERG) but negative for CK (Fig. 6f). Therefore, IHC performed to distinguish carcinoma from angiosarcoma should include both epithelial and endothelial markers [9, 10]. Based on the histological features and results of IHC, our patient was diagnosed with a malignant phyllodes tumor of the breast with an angiosarcoma component.

Occasionally, specific heterologous sarcomatous elements, including liposarcoma, osteosarcoma, chondrosarcoma, fibrosarcoma, and rhabdomyosarcoma, may be observed in phyllodes tumors [11]. Such malignant phyllodes tumors account for approximately 20% of all cases. The heterologous angiosarcoma component is considerably rare, and only four cases of angiosarcoma developing in the clinical setting of a phyllodes tumor have been reported [12–15]. Our case is the fifth case in the literature.

When the chest wall defect is over 300 cm^2^, free tissue transfer or multiple flaps are warranted [16]. Sometimes, prosthetic mesh should be used at the defect or weak part for reinforcement. Most cases are reconstructed successfully, but some have complications, such as flap loss, haematoma, infection, delayed wound healing, dehiscence or vascular thrombosis. However, cases with chest wall defect over 30 × 25 cm are rare. Joo Seok Park et al. [17] and Romain Bosc et al. [18] reported that only one case had chest wall defect over 700 cm^2^ in their studies. Increased size and complexity of chest wall resections could require advanced reconstructive techniques. The technique is still challenging and needs more experience.

TACE is used in locally advanced breast cancer for downstaging and increasing resectability [19]. TACE with superabsorbent polymer microspheres is a well-tolerated and feasible palliative option for patients with pulmonary or mediastinal metastasis from breast cancer [20]. For giant phyllodes tumors, only one case report demonstrated the effect of preoperative chemoembolization. Our case of a giant malignant phyllodes tumor is the second to undergo simple mastectomy without skin grafting through preoperative chemoembolization, which was useful in reducing tumor size. Treatment procedures were successful with no Common Terminology Criteria for Adverse Events (CTCAE) grade 3/4 toxicity observed. Laboratory data revealed no thrombocytopenia, hyperbilirubinemia, hypoleucocytosis, or failure of hepatic or renal functions. TACE causes postembolization syndrome, with localized pain, fever, vomiting, nausea, and fatigue being the commonest adverse events [21]. In our case, only fever and localized pain were noted after several days of TACE. Therefore, we confirm that TACE was highly effective in shrinking the giant phyllodes tumor in our patient and is a safe procedure.

In our study, the tumor had invaded the right pectoralis major. Chest wall invasion is an uncommon event. Reinfuss et al. reported 170 women with phyllodes tumors, 2.4% of cases with pectoralis major invasion [22]. Such cases were treated through radical resection with chest wall reconstruction [23]. Because breast CT after TACE indicated shrinkage of the right breast tumor, our patient did not undergo chest wall resection and reconstruction. However, tumor recurrence was discovered over the right chest wall 1 month after surgery. According to the literature, local recurrence rates after margin-negative breast-conserving resections of malignant phyllodes tumours are unacceptably high, at 23 to 30% [24]. Margin-negative resection combined with adjuvant radiotherapy is a very effective therapy for local control of malignant phyllodes tumours [25]. Therefore, early radiotherapy after surgery may be recommended for preventing recurrence.

In summary, initial surgical resection of giant phyllodes tumors is challenging. We recommend performing TACE prior to surgery. Preoperative TACE was effective and safe for our patient. If the tumor has invaded the chest wall, early radiotherapy after surgery may be recommended for preventing recurrence. Additional studies are required to establish and demonstrate the efficacy of this procedure.

## Data Availability

The datasets used and/or analyzed during the current study are available from the corresponding author on reasonable request.

## Abbreviations

CA153: Carcinoma Antigen 15–3
CD31: Cluster of differentiation 31, platelet endothelial cell adhesion molecule
CD34: Cluster of differentiation 34, hematopoietic progenitor cell antigen
CEA: Carcinoembryonic antigen
CK: Cytokeratin
CNB: Core needle biopsy
CT: Computed tomography
CTCAE: Common Terminology Criteria for Adverse Events
ERG: Erythroblast transformation-specific related gene
IHC: Immunohistochemistry
MRI: Magnetic resonance imaging
S100: S100 calcium binding protein
STAT6: Signal transducer and activator of transcription 6
TACE: Transcatheter arterial chemoembolization
